# Epidemiological Characteristics of HSV-1 and HSV-2 in 177,599 Patients Based on PCR Testing in South Korea (2018–2022)

**DOI:** 10.3390/pathogens14111107

**Published:** 2025-10-30

**Authors:** Hyeong Ho Kim, Sung Hun Jang, Jeong Su Han, Jae-Sik Jeon, Jae Kyung Kim

**Affiliations:** 1 Department of Biomedical Laboratory Science, College of Health Sciences, Dankook University, Cheonan-si 31116, Republic of Korea; hohyeongkim@dankook.ac.kr (H.H.K.); jshan1162@naver.com (J.S.H.); zenty87@naver.com (J.-S.J.); 2 Department of Medical Laser, Graduate School of Medicine, Dankook University, Cheonan-si 31116, Republic of Korea; well8143@naver.com

**Keywords:** age distribution, epidemiology, herpes simplex virus, herpes simplex virus type 1, herpes simplex virus type 2, sexually transmitted diseases, Sustainable Development Goals (SDG 3: good health and well-being)

## Abstract

HSV-1 is associated with oral lesions and non-sexual transmission; HSV-2 is primarily transmitted through sexual contact and causes genital infections. Understanding the epidemiological dynamics of both viruses is essential for guiding targeted public-health responses. We conducted a retrospective analysis of 177,599 clinical specimens collected between September 2018 and December 2022 from patients with symptoms suggestive of sexually transmitted infections (STIs) at healthcare institutions across South Korea. HSV-1 and HSV-2 were identified using a real-time PCR assay; positivity rates were stratified by age, sex, specimen type, and year of testing. The overall positivity rate was 0.26% for HSV-1 and 1.60% for HSV-2. HSV-1 was most prevalent among individuals aged <19 years; HSV-2 showed the highest positivity in females aged 20–29 years, declining with age thereafter. HSV-2 positivity was significantly higher in females than in males. A significant decline in HSV-2 positivity was observed over the 5-year study period, while HSV-1 positivity remained stable. This nationwide PCR-based study reveals distinct age- and sex-related epidemiological patterns of HSV-1 and HSV-2. The findings support the need for age-specific and gender-specific STI screening strategies and health education programs. The declining trend in HSV-2 infection may reflect the impact of recent public-health initiatives.

## 1. Introduction

Herpes simplex viruses (HSVs) types 1 and 2 are double-stranded DNA viruses of the *Simplexvirus* genus; they are widely distributed worldwide and of considerable clinical and public-health importance. HSV-1 is primarily transmitted through non-sexual contact and is associated with orolabial infections; HSV-2 is mainly transmitted through sexual contact and causes genital herpes [1,2,3]. Both viruses establish lifelong latency in sensory ganglia and can undergo periodic reactivation, resulting in recurrent disease that imposes substantial burdens of pain, psychological distress, and social stigma [4,5].

According to the World Health Organization (WHO), an estimated 3.7 billion people are infected with HSV-1 and approximately 418 million are infected with HSV-2, with disproportionately higher prevalence among women and individuals residing in low- and middle-income countries [4,6,7]. Notably, HSV-2 infection is recognized as a major risk factor for HIV acquisition and vertical transmission, leading to neonatal herpes and pregnancy-related complications [5,8,9].

Advances in molecular diagnostics have greatly improved HSV detection. In particular, real-time polymerase chain reaction (PCR) offers higher sensitivity and specificity than conventional culture or immunoassays and enables the rapid simultaneous detection of multiple pathogens, including HSV-1 and HSV-2 [2,10,11]. These advantages have positioned real-time PCR as a cornerstone in clinical diagnosis and in large-scale epidemiological investigations [11,12,13].

However, most previous epidemiological studies have been limited by small sample sizes or single-center data, with insufficient stratification by age and sex. In South Korea, large-scale population-based studies employing high-throughput molecular diagnostic data to characterize HSV epidemiology remain scarce, thereby limiting the understanding of HSV transmission dynamics in relation to demographic factors and evolving public health environments [14,15].

To address these gaps, we conducted a molecular diagnostics-based epidemiological study utilizing 177,582 real-time PCR test results accumulated over a 5-year period (2018–2022). By stratifying HSV-1 and HSV-2 positivity rates according to age, sex, year, and specimen type, this study provides measurable and reproducible evidence to inform sexually transmitted infection (STI) prevention strategies and targeted high-risk group screening. Furthermore, this study fills a long-standing gap in large-scale HSV epidemiological data, provides updated information to facilitate international comparisons and multicenter research, and offers actionable evidence to guide future public-health policy development in an era of expanding molecular diagnostic capacity.

## 2. Materials and Methods

### 2.1. Study Design and Data Source

This retrospective cross-sectional study analyzed 177,599 clinical specimens processed at U2Bio, a molecular diagnostic laboratory in Seoul, South Korea. Specimens were collected between September 2018 and December 2022 from patients presenting clinical symptoms suggestive of STIs, such as papules, pustules, or rashes. Sample types included swab specimens, urine, and tissue, which were obtained from hospitals and clinics nationwide.

### 2.2. Inclusion and Exclusion Criteria

The study population comprised both male and female patients, ranging in age from <19 years to >70 years, who underwent real-time PCR testing for HSV-1 and HSV-2 at U2Bio based on clinical suspicion of STI, as determined by the attending physician. Exclusion criteria were as follows: (1) specimens submitted for routine screening without clinical symptoms; (2) incomplete demographic information (e.g., missing age or sex); and (3) duplicate submissions, in which case only the first test result per patient was included.

### 2.3. Ethical Considerations

This study was conducted in accordance with the Declaration of Helsinki and was approved by the Institutional Review Board of Dankook University (Approval Code: 2023066). As this study used anonymized secondary data, informed consent was waived.

### 2.4. Nucleic Acid Extraction and PCR Testing

Specimens were stored at −70 °C until analysis. DNA was extracted using the ExiPrep Dx Bacteria Genomic DNA Kit (Bioneer, Daejeon, Republic of Korea) following the manufacturer’s instructions. HSV-1 and HSV-2 were detected with the AccuPower^®^ STI8B-Plex Real-Time PCR Kit on the Exicycler™ 96 system (Bioneer). PCR amplification was performed under the manufacturer’s recommended cycling conditions (initial denaturation at 95 °C for 5 min, followed by 45 cycles of 95 °C for 5 s and 55 °C for 5 s). Positive results were defined according to cycle threshold (Ct) values specified in the manufacturer’s interpretation guidelines, targeting the US4 gene for HSV-1 (111 bp) and the gG gene for HSV-2 (86 bp). This commercially available assay has been previously used in clinical studies for respiratory virus detection [15].

### 2.5. Statistical Analysis

Statistical analyses were performed using SPSS software, version 28.0 (IBM Corp., Armonk, NY, USA). Positivity rates for HSV-1 and HSV-2 were calculated and stratified by age, sex, year, and specimen type. Group comparisons of proportions were conducted using the chi-square test, and odds ratios (ORs) with 95% confidence intervals (CIs) were calculated. A two-sided *p*-value of <0.05 was considered statistically significant. In addition, to control for potential confounding factors, multivariable logistic regression analyses were performed to evaluate independent effects of sex, age group, and specimen type on HSV positivity. Adjusted odds ratios (aORs) with 95% CIs were derived from these models. Furthermore, regression-based temporal trend analyses were conducted to assess annual changes in HSV-1 and HSV-2 positivity between 2018 and 2022 while adjusting for sex. Results with two-sided *p*-value of <0.05 were considered statistically significant. Use of Artificial Intelligence Tools: During the preparation of this manuscript, the authors used ChatGPT (OpenAI, San Francisco, CA, USA) solely for English language refinement and style improvement. The authors thoroughly reviewed and verified all AI-assisted text to ensure accuracy, consistency, and scientific integrity. No AI tools were used for data analysis, interpretation, or generation of scientific content.

## 3. Results

Of 177,582 clinical specimens, the overall positivity rate was 0.26% for HSV-1 and 1.61% for HSV-2, indicating a substantially higher burden of HSV-2 infection in this population (Table 1). This pattern was consistently observed across various demographic strata.

### 3.1. Age-Specific Distribution

**Figure 1:**
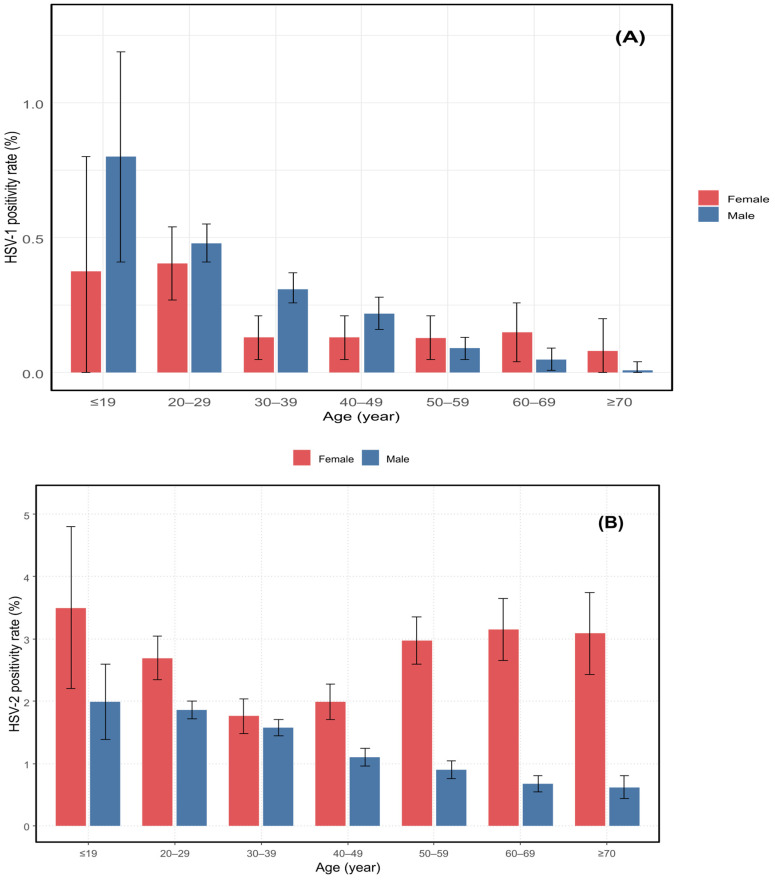
HSV-1 and HSV-2 positivity rates by age and sex. Positivity rates are shown as percentages of total tested individuals within each age group. Blue and red bars represent males and females, respectively. Panel (**A**) shows HSV-1 positivity rates; panel (**B**) shows HSV-2 positivity rates across seven age groups.

**Figure 2:**
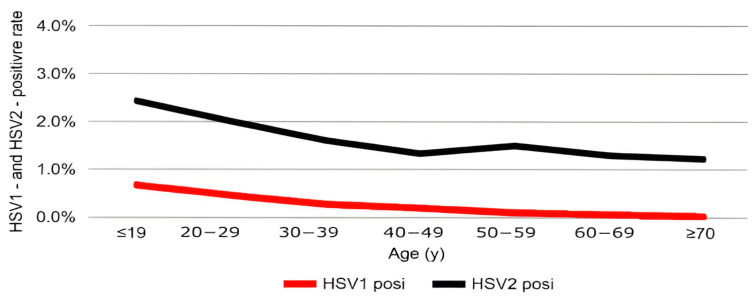
Positivity rates of HSV-1 and HSV-2 by age group. Red and black lines indicate HSV-1 and HSV-2 positivity rates (%), respectively, across seven age groups (≤19, 20–29, 30–39, 40–49, 50–59, 60–69, and ≥70 years).

The positivity rate of HSV-1 infection was highest in individuals aged <19 years (0.68%) and gradually declined with increasing age. Similarly, HSV-2 positivity peaked in patients under 19 years of age (2.42%) and showed a continuous decrease across older age groups (Table 1). These age-related patterns are presented graphically to highlight their clinical significance (Figure 1A,B and Figure 2).

### 3.2. Sex-Related Differences in Positivity Rates

**Figure 3:**
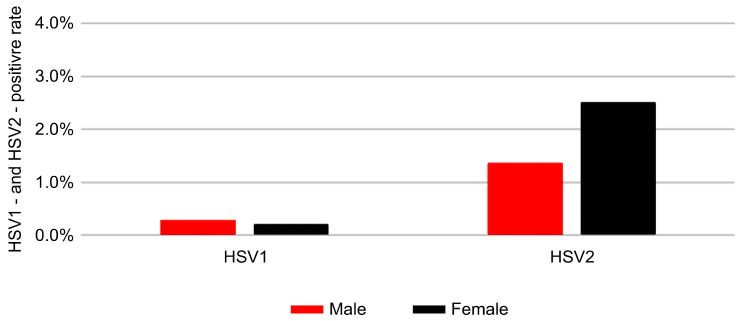
Positivity rates of HSV-1 and HSV-2 by sex. Red and black bars represent positivity rates (%) for males and females, respectively.

A significant sex-related difference was observed for HSV-2, with higher positivity in women (2.50%) than in men (1.37%) (*p* < 0.001; Table 2). This difference was consistent across age groups and was particularly pronounced among young women. Similarly, HSV-1 prevalence also showed a statistically significant sex-related difference, with positivity rates of 0.2% in women and 0.3% in men (*p* = 0.003). The sex-specific positivity trends are illustrated in Figure 3.

To evaluate whether these sex differences were influenced by potential confounding factors, an additional multivariable analysis was performed after adjusting for age group and specimen type. After adjustment, female sex was no longer significantly associated with HSV-2 positivity (aOR = 1.00, 95% CI: 0.88–1.13, *p* = 0.969), indicating that the higher unadjusted positivity in women may be explained by differences in age distribution and specimen characteristics. In contrast, swab specimens showed a significantly higher likelihood of HSV-2 detection compared with urine samples (aOR = 2.30, 95% CI: 2.01–2.62, *p* < 0.001). For HSV-1, however, even after controlling for the same factors, female sex remained significantly less associated with positivity than male sex (aOR = 0.14, 95% CI: 0.10–0.19, *p* < 0.001); specimen type did not have a significant effect (*p* = 0.478) (Table 3).

### 3.3. Temporal Trends from 2018 to 2022

**Figure 4:**
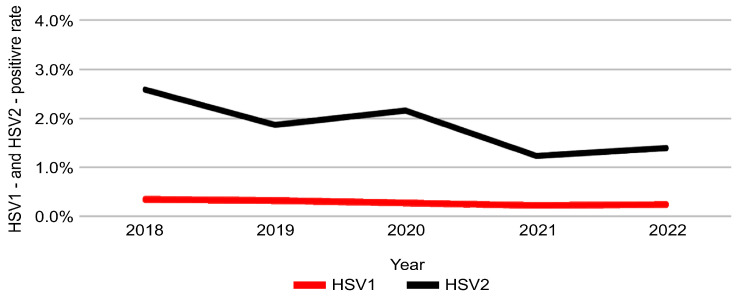
Annual positivity rates of HSV-1 and HSV-2 during 2018–2022. Red and black lines represent the yearly positivity rates (%) for HSV-1 and HSV-2, respectively, during the study period.

The positivity rate of HSV-2 declined significantly over the 5-year study period, decreasing from 2.61% in 2018 to 1.40% in 2022 (*p* < 0.001) (Table 4). In contrast, HSV-1 positivity remained stable during the same period, with no statistically significant change (*p* = 0.065). These temporal trends are illustrated in Figure 4.

However, regression-based temporal trend analysis revealed a significant annual decrease in HSV-2 positivity (aOR = 0.86; 95% CI = 0.83–0.88; *p* < 0.001) and a modest but significant decline in HSV-1 positivity (aOR = 0.90; 95% CI = 0.84–0.98; *p* = 0.009), suggesting a sustained downward trend in both viral infections during 2018–2022 (Table 5).

### 3.4. Distribution by Specimen Type

**Figure 5:**
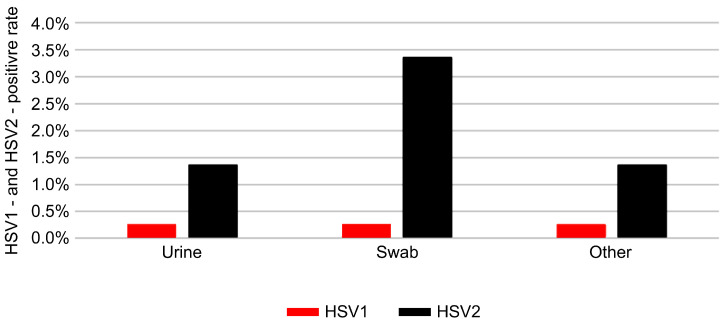
Positivity rates of HSV-1 and HSV-2 by specimen type. Red and black bars show positivity rates (%) of HSV-1 and HSV-2, respectively, across urine, swab, and other specimen categories.

Among the different types of clinical specimens, HSV-2 was most frequently detected in swab samples, which are commonly collected from mucocutaneous lesions. Urine and tissue specimens demonstrated relatively lower positivity rates, consistent with clinical expectations regarding specimen suitability for HSV detection. The distribution of HSV positivity across specimen types is shown in Figure 5. The total number of tests (n) and sex distribution for each specimen type are presented in Table 6. Among all specimens, urine samples were predominantly obtained from men, whereas swab samples were mainly collected from women, reflecting differences in clinical testing patterns by infection site and patient demographics.

## 4. Discussion

This large-scale retrospective study analyzed 177,599 clinical specimens collected across South Korea to investigate the epidemiological patterns of HSV-1 and HSV-2 using real-time PCR. The findings delineate age-specific, sex- specific, and time-specific trends and provide critical implications for public-health strategies in HSV management.

A key observation was the distinct age-related distribution of HSV-1 and HSV-2 infections. HSV-1 positivity was highest among individuals aged <19 years, consistent with global reports that HSV-1 transmission predominantly occurs during childhood through non-sexual, close-contact interactions [1,4,12,16]. Similarly, HSV-2 positivity was also highest in individuals aged <19 years; however, the absolute number of positive cases peaked in those in their twenties. This pattern likely reflects the onset of sexual activity and increased susceptibility influenced by both biological and social factors [6,7,8]. These results emphasize the necessity of tailored prevention programs and early screening strategies, particularly targeting individuals in their twenties.

The study also confirmed significant sex-specific differences in HSV-2, with consistently higher positivity rates among females than among males. This disparity aligns with previous research suggesting contributions from differences in genital mucosal susceptibility, hormonal influences, sexual behavior, and healthcare-seeking patterns [5,11]. These findings underscore the importance of sex-specific public health interventions and improved diagnostic accessibility for women.

Another noteworthy finding was the significant decline in HSV-2 positivity between 2018 and 2022. This trend may be attributable to increased awareness of sexually transmitted infections (STIs), enhanced sexual health education, and greater access to diagnostic services [4,17,18]. In addition, behavioral changes associated with the COVID-19 pandemic, such as reduced sexual contact due to social distancing, may also have contributed. In contrast, HSV-1 positivity remained relatively stable in the descriptive analysis, although regression-based modeling indicated a modest but statistically significant annual decline, suggesting that interventions targeting sexual transmission had limited impact on HSV-1 infections, which are primarily transmitted through non-sexual routes. When compared with trends in international data, the declining trend of HSV-2 positivity and the relative stability of HSV-1 observed in this study are largely consistent with global reports. Studies from the USA and Europe have similarly shown a gradual reduction in HSV-2 prevalence, mainly attributed to increased sexual health awareness and safer behavioral practices [19,20]. In contrast, HSV-1 remains highly prevalent worldwide, particularly in low- and middle-income regions, where non-sexual transmission during childhood continues to dominate [21,22,23]. However, the relatively higher HSV-2 burden among young Korean women suggests that regional differences in sexual behavior, healthcare accessibility, and sociocultural norms influence transmission dynamics. These findings highlight the need for tailored public-health strategies that consider both global trends and local epidemiological contexts.

Although this study did not include information on anatomical sites or clinical manifestations, which precluded distinguishing genital HSV-1 infections from non-genital cases, the relatively higher HSV-1 positivity among younger individuals may indirectly suggest behavioral changes consistent with global observations, such as increased orogenital contact. Several recent studies have reported a rising proportion of genital herpes cases attributed to HSV-1 rather than HSV-2, particularly among younger women, reflecting evolving sexual behaviors [22,24]. This limitation has been acknowledged to provide the appropriate context for interpreting these findings.

The analysis by specimen type revealed that HSV-2 positivity was highest in swab samples, reflecting the suitability of mucosal or lesion-based specimens for detecting active viral shedding. In contrast, urine and tissue specimens demonstrated lower positivity rates, likely owing to reduced viral loads and differences in anatomical sampling sites. These findings reinforce current clinical recommendations to prioritize swab specimens for HSV diagnostics in order to optimize detection accuracy. However, the preferential use of swab specimens, particularly among female patients, may also contribute to the observed sex-specific differences in HSV-2 detection.

Although this study focused solely on HSV-1/2 infections, Korean surveillance data indicate that other sexually transmitted infections, including those of *Chlamydia trachomatis*, *Neisseria gonorrhoeae*, *Mycoplasma genitalium*, and *Trichomonas vaginalis*, show similar epidemiological patterns, with the highest detection rates observed among women in their twenties [25]. These similarities indicate that common behavioral and biological factors may underlie STI transmission dynamics. Integrative studies using multiplex PCR data encompassing multiple pathogens would be valuable for elucidating potential co-infection patterns and establishing comprehensive STI control strategies.

The principal strength of this study lies in its scale and representativeness, leveraging extensive real-time PCR data from diverse regions and multiple institutions. The use of real-time PCR ensured high diagnostic sensitivity and specificity, which are particularly valuable for symptomatic patients, thereby enabling robust epidemiological assessments [2,10,11,13].

Nevertheless, several limitations should be acknowledged. First, real-time PCR detects viral DNA at a single time point, reflecting current viral shedding rather than cumulative lifetime exposure or prior infection history. Thus, it may underestimate the total burden of HSV infection, which would be better captured by serological testing. In particular, serology would provide additional insight into the prevalence of asymptomatic HSV-1 infections. Second, the study population was limited to symptomatic individuals seeking medical care, which may have resulted in an overestimation of positivity rates compared with those in asymptomatic or general populations. Third, owing to the retrospective nature of the dataset, detailed clinical information such as symptom severity, sexual behavior, contraceptive practices, and vaccination history was unavailable, limiting interpretation of risk factors.

Despite these limitations, this study provides valuable, data-driven evidence to inform public-health policy. The results strongly support the implementation of age-specific and sex-specific screening programs, targeted sexual-health education, and improved diagnostic accessibility, particularly for women who bear the highest burden of HSV-2 infection. Future research should incorporate longitudinal cohort designs, detailed clinical assessments, sexual behavior surveys, and serological data to clarify determinants of HSV transmission and optimize prevention strategies [26].

In summary, this study highlights the importance of molecular-diagnostic-based surveillance for HSV and offers robust evidence to guide future public-health planning. The distinctive epidemiological patterns of HSV-1 and HSV-2 identified here demonstrate the necessity of age-specific and sex-specific management strategies, which may serve as a motivation for strengthening STI surveillance systems and facilitating international comparative research.

## 5. Conclusions

This nationwide PCR-based analysis of 177,599 clinical specimens provides population-level evidence on the epidemiological dynamics of HSV-1 and HSV-2 in South Korea. HSV-2 showed higher positivity in women and a significant decline over the study period, whereas HSV-1 remained stable and was most prevalent in younger age groups. These findings emphasize the need for age-specific and sex-specific screening strategies, improved diagnostic accessibility, and targeted sexual-health education. The large-scale molecular data presented here offer a solid evidence base for strengthening STI surveillance and informing public-health policies, while also serving as a valuable reference for international comparative research.

## Figures and Tables

**Table 1:** Age-specific positivity rates of HSV-1 and HSV-2 in 177,599 clinical specimens collected from 2018 to 2022.

Age (Years)	≤19 (n = 2801)	20–29 (n = 43,877)	30–39 (n = 43,793)	40–49 (n = 32,072)	50–59 (n = 26,705)	60–69 (n = 18,674)	≥70 (n = 9677)	χ^2^	*p*-Value
HSV-1, n (%) (95% CI)	19 (0.68) (0.37–0.98)	205 (0.47) (0.40–0.53)	122 (0.28) (0.23–0.33)	63 (0.20) (0.15–0.24)	27 (0.10) (0.06–0.14)	14 (0.07) (0.04–0.11)	3 (0.03) (0.00–0.07)	170.41	<0.001 ***
HSV-2, n (%) (95% CI)	68 (2.42) (1.86–3.00)	884 (2.01) (1.88–2.15)	705 (1.60) (1.49–1.73)	430 (1.34) (1.21–1.47)	401 (1.50) (1.36–1.65)	242 (1.29) (1.13–1.46)	120 (1.24) (1.02–1.46)	94.11	<0.001 ***

This table summarizes the distribution of HSV-1 and HSV-2 positivity rates across different age groups, showing a higher HSV-1 detection in younger individuals and peak HSV-2 detection in individuals in their 20s. HSV, herpes simplex virus; CI, confidence interval *** < 0.001.

**Table 2:** Positivity rates of HSV-1 and HSV-2 by sex from 2018 to 2022.

Sex	Male, n (%) (n = 137,027)	Female, n (%) (n = 40,572)	χ^2^	*p*-Value
HSV-1	376 (0.3)	77 (0.2)	8.809	0.003 *
HSV-2	1837 (1.37)	1015 (2.50)	267.950	<0.001 ***

A significantly higher HSV-2 positivity rate is observed in females than in males, with the greatest difference seen in young adult women. HSV, herpes simplex virus. * < 0.005 *** < 0.001.

**Table 3:** Adjusted odds ratios (aORs) for HSV-1 and HSV-2 positivity according to sex and specimen type.

Variable	Category	Reference	aOR	95% CI	*p*-Value
HSV-2					
Sex	Female	Male	1000	0.88–1.13	0.969
Specimen type	Swab	Urine	2.30	2.01–2.62	<0.001
	Other	Urine	0.88	0.75–1.03	0.109
Age group	30–39	≤19	0.69	0.53–0.88	0.003
	40–49	≤19	0.55	0.43–0.72	<0.001
	50–59	≤19	0.64	0.50–0.84	<0.001
	60–69	≤19	1.3	0.99–1.71	0.062
	≥70	≤19	0.55	0.41–0.75	<0.001
HSV-1					
Sex	Female	Male	0.14	0.10–0.19	<0.001
Specimen type	Swab	Urine	0.88	0.61–1.26	0.478
	Other	Urine	0.19	0.13–0.28	<0.001
Age group	20–29	≤19	5.81	3.59–9.39	<0.001
	30–39	≤19	0.36	0.22–0.59	<0.001
	40–49	≤19	0.27	0.16–0.45	<0.001
	50–59	≤19	0.8	0.44–1.47	0.476
	60–69	≤19	0.51	0.24–1.10	0.087
	≥70	≤19	0.05	0.01–0.16	<0.001

Multivariable logistic regression analysis of HSV-1 and HSV-2 positivity adjusted for sex, age group, and specimen type. Reference categories were Male for sex, ≤19 years for age group, and urine for specimen type. Abbreviations: aOR, adjusted odds ratio; CI, confidence interval; HSV, herpes simplex virus.

**Table 4:** Annual positivity rates of HSV-1 and HSV-2 from 2018 to 2022.

Year	2018 (n = 5912)	2019 (n = 25,394)	2020 (n = 33,885)	2021 (n = 42,764)	2022 (n = 69,644)	χ^2^	*p*-Value
HSV1, n (%)	20 (0.3)	80 (0.3)	94 (0.3)	94 (0.2)	165 (0.2)	8.855	0.065
HSV2, n (%)	153 (2.6)	474 (1.9)	731 (2.2)	524 (1.2)	968 (1.4)	170.083	<0.001 ***

HSV, herpes simplex virus. *** <0.001.

**Table 5:** Temporal trend analysis of HSV-1 and HSV-2 positivity (2018–2022).

Virus	Statistical Model	Adjusted Odds Ratio (aOR) per Year	95% Confidence Interval	*p*-Value
HSV-2	Logistic regression (per 1-year increase)	0.86	0.83–0.88	< 0.001
HSV-1	Logistic regression (per 1-year increase)	0.9	0.84–0.98	0.009

The model was fitted using binomial logistic regression with year as a continuous variable. Abbreviations: HSV, herpes simplex virus; aOR, adjusted odds ratio; CI, confidence interval.

**Table 6:** Distribution of total tests and positive cases for HSV-1 and HSV-2 by specimen type, age group, and sex (2018–2022).

Sex	Age (Years)	Specimen Type	Total Tests (n)	HSV-1 Positive (n)	HSV-2 Positive (n)
Male	≤19	Urine	1815	11	36
		Swab	25	1	2
		Other	161	4	2
	20–29	Urine	33,334	153	582
		Swab	232	4	32
		Other	2185	15	51
	30–39	Urine	32,722	100	484
		Swab	216	6	35
		Other	2448	5	38
	40–49	Urine	21,596	48	227
		Swab	120	1	16
		Other	1937	3	19
	50–59	Urine	17,032	15	139
		Swab	88	0	15
		Other	1856	2	19
	60–69	Urine	12,505	7	78
		Swab	31	0	9
		Other	1474	0	8
	≥70	Urine	6666	0	36
		Swab	14	0	6
		Other	570	1	3
Female	≤19	Urine	308	1	6
		Swab	461	2	22
		Other	31	0	0
	20–29	Urine	3288	15	64
		Swab	4572	17	151
		Other	266	3	4
	30–39	Urine	2473	1	33
		Swab	5637	10	111
		Other	297	0	4
	40–49	Urine	2920	5	48
		Swab	5039	6	112
		Other	460	0	8
	50–59	Urine	3825	3	102
		Swab	3333	4	121
		Other	571	1	7
	60–69	Urine	2820	4	66
		Swab	1520	3	75
		Other	324	0	6
	≥70	Urine	1739	1	43
		Swab	591	1	29
		Other	97	0	3

Detailed distribution of total specimens tested and positive cases for HSV-1 and HSV-2 by specimen type, age group, and sex. Urine, swab, and other specimens include samples submitted for multiplex PCR testing between 2018 and 2022.

## Data Availability

The data that support the findings of this study are derived from patient records at U2Bio and are subject to ethical and legal restrictions. Owing to privacy and confidentiality concerns, the raw datasets cannot be made publicly available. However, anonymized summary data may be obtained from the corresponding author upon reasonable request, subject to approval by the Institutional Review Board.

## Abbreviations

The following abbreviations are used in this manuscript:

CI	Confidence Interval
HSV	Herpes Simplex Virus
HSV-1	Herpes Simplex Virus 1
HSV-2	Herpes Simplex Virus 2
OR	Odds Ratio
Ct	Threshold Cycle
WHO	World Health Organization
